# Ionic gold demonstrates antimicrobial activity against *Pseudomonas aeruginosa* strains due to cellular ultrastructure damage

**DOI:** 10.1007/s00203-021-02270-1

**Published:** 2021-03-29

**Authors:** Miguel Reyes Torres, Anthony J. Slate, Steven F. Ryder, Maliha Akram, Conrado Javier Carrascosa Iruzubieta, Kathryn A. Whitehead

**Affiliations:** 1 Microbiology at Interfaces, Manchester Metropolitan University, Chester Street, Manchester, M1 5GD UK; 2 Department of Biology and Biochemistry, University of Bath, Claverton Down, Bath, BA2 7AY UK; 3 Department of Animal Pathology, Animal Production, and Food Science and Technology, Universidad de Las Palmas de Gran Canarias, Gran Canarias, 35413 Arucas, Spain

**Keywords:** Ionic gold, Metals, Resistance, *Pseudomonas aeruginosa*, Antimicrobials, Mechanism of action

## Abstract

Due to the ever-increasing rise of antimicrobial resistant (AMR) bacteria, the development of alternative antimicrobial agents is a global priority. The antimicrobial activity of ionic gold was explored against four *Pseudomonas aeruginosa* strains with different AMR profiles in order to determine the antimicrobial activity of ionic gold and elucidate the mechanisms of action. Disc diffusion assays (zone of inhibition: ZoI) coupled with minimum inhibitory/bactericidal concentrations (MIC/MBC) were conducted to determine the antimicrobial efficacy of ionic gold. Scanning electron microscopy (SEM) was used to visualise morphological changes to the bacterial cell ultrastructure. Strains with increased AMR were slower to grow which is likely a fitness cost due to the enhanced AMR activity. Although greater concentrations of ionic gold were required to promote antimicrobial activity, ionic gold demonstrated similar antimicrobial values against all strains tested. Lowry assay results indicated that protein leakage was apparent following incubation with ionic gold, whilst SEM revealed cellular ultrastructure damage. This study suggests that the application of ionic gold as an alternative antimicrobial is promising, particularly against AMR *P. aeruginosa*. The antimicrobial activity of ionic gold against *P. aeruginosa* could potentially be utilised as an alternative therapeutic option in wound management, an approach that could benefit healthcare systems worldwide.

## Introduction

The emergence of antimicrobial resistance (AMR) is a major global issue, due to the persistent overuse of antimicrobials, such as antibiotics in medicine and agriculture (Blair et al. 2015; Slate et al. 2019). Antimicrobial resistance can be defined as the ability of a microorganism to resist growth inhibition (and/or death) exerted by an antimicrobial, beyond the normal susceptibility of the microbial species (Verraes et al. 2013). In parallel to an increase in patient morbidity and mortality, AMR has placed a huge financial burden on healthcare services (Lowy 2003; Bassetti et al. 2013; Breathnach 2013). The number of deaths caused by multidrug-resistant (MDR) bacteria worldwide is estimated to be approximately 700,000 deaths per annum (Tillotson and Zinner 2017). Furthermore, it is estimated that by 2050, mortality rates due to AMR infections will exceed 10 million people per year; superseding cancer as the leading cause of mortality worldwide (O’Neill 2018). The lack of effective therapies to combat AMR infections has resulted in an urgent need to develop alternative antimicrobial therapies (Ventola 2015).

*Pseudomonas aeruginosa* is a Gram-negative, rod-shaped, facultative anaerobic bacterium (Wilson and Dowling 1998). Owing to its remarkable metabolic versatility, it is able to assimilate a wide range of compounds, allowing survival and proliferation in low nutrient and anaerobic environments (Arai 2011; Wu et al. 2015). This versatility facilitates pathogenesis as it enables the bacteria to adapt to different microenvironments. This bacterium is an opportunistic pathogen, often causing infections in burns and immunocompromised patients; it is the most prevalent organism isolated in cystic fibrosis patients (Fichtenbaum et al. 1994; Lyczak et al. 2000; Hart and Winstanley 2002; Turner et al. 2014; Winstanley et al. 2016). *Pseudomonas aeruginosa* is a major cause of nosocomial infections and it is a major contributor of chronic wound infections (Karaky et al., 2020). In addition, *P. aeruginosa* biofilms are particularly problematic due to the development of persister cells which can render traditional antibiotic therapies ineffective (Mulcahy et al. 2014). For example, chronic wounds in diabetics patients are notoriously difficult to treat, due to the overwhelming presence of drug-tolerant persister cells (Dowd et al. 2011).

The use of metals in medical applications is an ancient practice (Lemire et al. 2013). Today, metals used in modern medicine as therapeutic agents include chemotherapy drugs, anti-arthritis drugs and antimicrobial agents (Mcquitty 2014). Different metals and their metallic states (*e.g.* solid, metal ions/oxides, nanoparticles) have different antimicrobial efficacies. Due to the non-specificity of metals and their ability to target multiple bacterial sites simultaneously, the acquisition of antimicrobial resistance is deemed low (Zhou et al. 2012; Lemire et al. 2013; Southam et al. 2017). Multiple applications have been described for gold nanoparticles, such as image detection and a carrier to transport drugs into cells (Yeh et al. 2012). Furthermore, ionic gold has previously demonstrated antimicrobial activity in both Au^1+^ and Au^3+^ variants (Shareena Dasari et al. 2015; Vaidya et al. 2017).

The aim of this study was to determine the antimicrobial activity of ionic gold against a range of *P. aeruginosa* type strains and clinical isolates with different antibiotic resistant profiles and to further elucidate the antimicrobial mechanisms of action.

## Materials and methods

### Bacterial strains and cultures

The bacterial strains utilised in this study included *Pseudomonas aeruginosa* (PAO1), *P. aeruginosa* (ATCC 9027) and two clinical *P. aeruginosa* isolates recovered from Manchester Royal Infirmary (MRI), (Manchester, UK) and Bolton Royal Hospital (RBH), (Bolton, UK). The strains were cultured using Tryptone Soya Agar (TSA) and Tryptone Soya Broth (TSB) (Oxoid, UK), which were prepared in accordance with the manufacturer’s instructions. The bacterial strains were streaked onto TSA and incubated at 37 ºC for 18 h. A single colony was transferred to 10 mL of TSB and incubated at 37 °C for 18 h in an orbital shaker (200 rpm) (GIO gyratory shaker, USA).

### Antibiotics and ionic gold

Characterization of the antimicrobial activity of ionic gold was performed with the Gold Standard for AAS (atomic absorption spectroscopy) (Sigma-Aldrich, UK), which comprises of gold, dissolved in a 5% (v/v) HCl solution, with trace amounts of HNO_3_ present, at a concentration of 1000 μg mL^−1^. The presence of nitric acid acts as a powerful oxidising agent to form Au^3+^ ions, whilst the presence of HCl provides chloride ions which promote the formation of chloroaurate anions (AuCl_4_^−^), this process is one of the most commonly employed methods in which gold is dissolved and the leaching reagent (the combination of HCl and HNO_3_) is known as *aqua regia* (Rao et al. 2020; Bae et al. 2020). The antibiotics included in this study were 5 µg ciprofloxacin, 30 µg ceftazidime and 30 µg gentamicin (Sigma-Aldrich, UK) for diffusion assays. Whilst, solutions of these antibiotics were prepared at a maximum concentration of 2048 μg mL^−1^ in sterile distilled water, except ciprofloxacin, which was dissolved in 2% (v / v) acetic acid for minimum inhibitory/bactericidal concentration (MIC/MBC) assays. The antibiotics were stored at 4 °C prior to use.

### Growth curves

Growth curves were prepared in 96-well, flat-bottomed culture plates (Sarstedt, USA), 200 µL of the starting inoculum was added, resulting in a starting culture of 0.05 OD_600 nm_ in each well, which equated to *ca.* 0.5 × 10^8^ CFU mL^−1.^ Growth curve data (*i.e.* absorbance values) was recorded using a SPECTROstar Nano multiplate reader (UK), into which, the plate was inserted and maintained at 37 ºC with agitation at 120 rpm. Measurements were recorded automatically by determining the absorbance of each sample (OD_600 nm_) every 10 min for 22 h. For each strain, triplicate growth curves were performed (*n* = 3).

### Antimicrobial susceptibility

#### Antimicrobial diffusion assays

*Pseudomonas aeruginosa* cultures were inoculated into TSB and incubated at 37 °C overnight with constant shaking (200 rpm). The cultures were diluted to an optical density (OD_600 nm_) of 0.025. One hundred microliters of the bacterial culture were pipetted onto TSA and spread across the agar. A commercial antibiotic disc ring device was placed onto the pre-inoculated agar (MAST, UK; M26/NCE) which was dried for 1 h and incubated overnight at 37 °C. After incubation, the diameter of each zone of inhibition (ZoI) was measured using electronic callipers (*n* = 3). The commercial M26/NCE disk contained the following antibiotics, 25 µg ampicillin, 50 µg chloramphenicol, 100 µg colistin Sulphate, 30 µg kanamycin, 30 µg nalidixic acid, 50 µg nitrofurantoin, 25 µg streptomycin and 100 µg tetracycline.

For the determination of the antimicrobial efficacy of the specific antibiotics against *P. aeruginosa*, the method was carried out as above except that 10 μL of 5 µg ciprofloxacin, 30 µg ceftazidime and 30 µg entamicin (Sigma-Aldrich, UK) and the ionic gold were added to sterile, antibiotic-free 6 mm diffusion discs (Sigma-Aldrich, UK) which were deposited onto the surface of the agar. As 5% HCl was used as the diluent for the gold, this was also tested and the observed ZoI was subtracted from the final ionic gold ZoI value. The inoculated plates were incubated and measured as above (*n* = 3). The antibiotics were dissolved in sterile distilled water except for ciprofloxacin, which was dissolved in 2% (v/v) acetic acid.

#### Minimum inhibitory concentration (MIC)

To determine the minimum inhibitory concentration (MIC), the strains were inoculated in TSB medium for 18 h at 37 °C. Bacterial cultures were centrifuged at 1721 × *g*, and the supernatant was removed,* P. aeruginosa* was normalised to an optical density of 0.05 at OD _600 nm_ in double-strength TSB, which equated to *ca.* 0.5 × 10^8^ CFU mL^−1^. The antibiotics were dissolved to a final concentration of 2048 μg mL^−1^. In a 96 well plate, the outer wells were filled with 200 µL sterile TSB. In the second column of wells, 200 µL of the antimicrobials at the maximum concentration and 100 μL of sterile distilled water was added to the sequential columns. A twofold dilution was carried out, 100 μL of the solution was transferred into the next column (column three); this process was repeated until column ten, where 100 µL of the solution was discarded ensuring all wells had a 100 µL total volume. Normalised *P. aeruginosa * solutions were combined with 0.15% triphenyl blue chloride (TBC) (this was included as a metabolic activity indicator). Aliquots of 100 μL of the bacterial suspension was then added to each well, giving a final volume of 200 μL. To avoid evaporation, the plate was closed and sealed with Parafilm^®^. The outer wells that contained sterile TSB served as the negative control. Column eleven contained *P. aeruginosa* but no antimicrobial (positive control). The plates were incubated at 37 °C for 18 h without agitation. This study was also conducted with the solvents the antimicrobials were dissolved in to ensure that the antimicrobial activity was not produced solely by the solvents. Following incubation, the MIC of the antimicrobial was defined as the minimum concentration that inhibited visible growth in that well, compared with the positive and negative controls utilised in this study (*n* = 3).

#### Minimum bactericidal concentration (MBC)

To determine the minimum bactericidal concentration (MBC), 25 μL of solution was taken from the last well (and the two concentrations above and below this) that demonstrated growth inhibition. The culture was spread across TSA and dried at room temperature for 1 h whilst maintaining aseptic conditions, before being incubated at 37 °C for 18 h without agitation. Following incubation, the lowest concentration that exhibited no growth was considered the MBC (*n* = 3).

#### Determination of extracellular proteins

To quantify the concentration of proteins released into the extracellular media, 100 mL of TSB was inoculated with *P. aeruginosa* and incubated at 37 °C for 18 h with constant agitation (200 rpm). The bacterial suspensions were centrifuged at 1721 × *g* for 7 min and replaced with PBS, this washing step was repeated twice before cultures were normalised to 1.00 OD_600 nm_. An aliquot of 2 mL of each sample was taken and added to the predetermined MIC concentration of the antimicrobial. A 2 mL aliquot was simultaneously treated with cetyltrimethylammonium bromide (CTAB) at the predetermined MIC concentration, which was established as the positive control. A third aliquot of the same volume, but untreated, was used as a negative control. The samples were incubated for 2 h at 37 °C and centrifuged at 10,625 × *g* for 15 min. The supernatant was collected and a Lowry assay for protein determination was performed (Lowry et al. 1951; Olson and Markwell 2007).

### Lowry method

The supernatant was diluted into 1:50, 1:200 and 1:1000 dilutions using sterile PBS. Aliquots of 5 mL of extemporaneous Lowry reagent (50 mL of alkaline reagent + 0.5 mL of copper reagent (Lowry et al. 1951) were added, vortexed for 10 s and incubated at room temperature for 10 min. Folin–Cicocalteau reagent (0.5 mL diluted in the same volume of deionised water) was added to the mixture and was left to stand for 30 min. The absorbance of the sample was measured in a spectrophotometer (Jenway, UK) at OD_660 nm_. The results were expressed as percentage protein release. For the quantification of the protein (mg), a calibration curve was performed utilising increasing known concentrations of bovine serum albumin (BSA) at 0 μg mL^−1^, 0.5 μg mL^−1^, 2.5 μg mL^−1^, 5 μg mL^−1^, 10 μg mL^−1^, 15 μg mL^−1^ and 20 μg mL^−1^.

### Scanning electron microscopy (SEM)

All *P. aeruginosa* strains were inoculated in 10 mL TSB and incubated overnight at 37 °C with constant shaking (200 rpm). After incubation, the optical density of the bacterial suspension was adjusted to 0.5 (OD_600 nm_) and treated with ionic gold at the predetermined MIC concentration, for 2 h and 18 h. An untreated aliquot was used as a negative control. The cultures were centrifuged for 7 min at 1721 × *g* and washed with PBS. Aliquots of 25 μL of culture were placed on sterile stainless steel (316L) (1 cm × 1 cm) and dried for 1 h at room temperature. The samples were immersed in 4% glutaraldehyde (Agar Scientific, UK) at 4 ºC for 18 h. The samples were dehydrated by sequential submersion in an ethanol gradient of increasing concentrations (10%, 30%, 50%, 70%, 90% and absolute ethanol). The dehydrated samples were placed onto carbon tabs (Agar Scientific, UK). Samples were dried by desiccation over 24 h and were sputter coated with gold (Polaron, UK) for 30 s (parameters: power 5 mA, 30 s, 800 V, vacuum 0.09 mbar, argon gas) prior to imaging using a JEOL JSM 5600LV scanning electron microscope.

### Statistical analysis

All experiments were performed in triplicate (*n* = 3). The error bars shown in the graphs correspond to the standard error of the mean. Statistical analysis of the data was carried out using Prism 8 (Version 8.4.3), following Shapiro–Wilk normality analysis, significant differences were determined at *p* < 0.05 using two-way ANOVAs coupled with Tukey’s tests for post hoc analysis. Asterisks denote significance, *p ≤ 0.05, **p ≤ 0.01, ***p ≤ 0.001 and ****p ≤ 0.0001.

## Results

### Bacterial growth curves

**Fig. 1:**
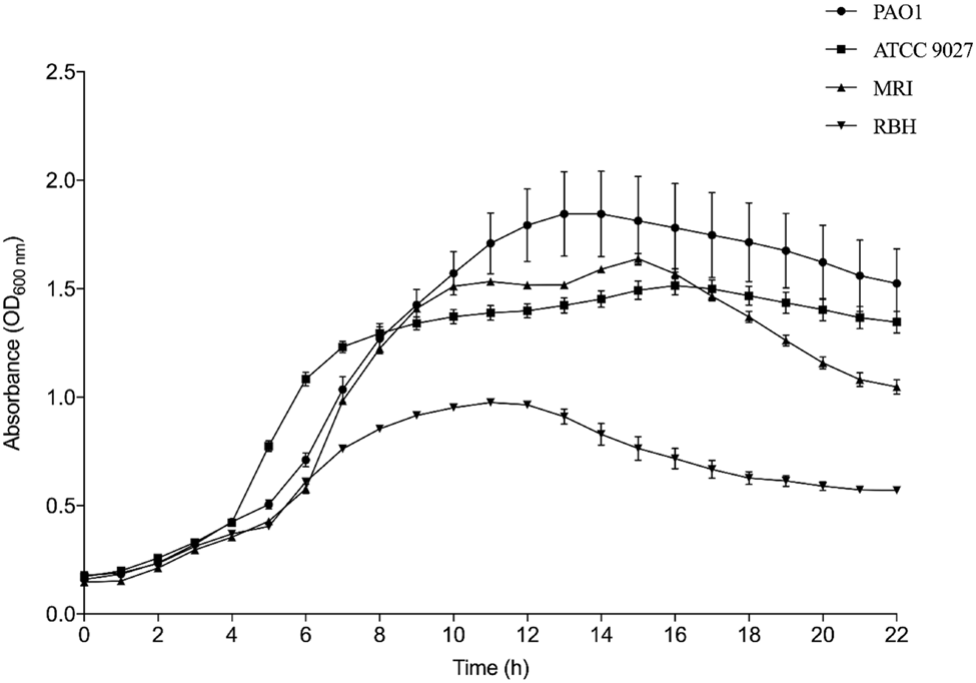
Growth kinetics of the four *P. aeruginosa* strains, OD_600 nm_ (conditions: TSB, 37 °C, 120 rpm agitation) was recorded every 10 min for 24 h (*n* = 3)

Growth curves were conducted for all the *P. aeruginosa* strains utilised in this study (Fig. 1). The growth curves obtained demonstrated a traditional four stage growth curve with lag, log (exponential), stationery and death phases observed. At 4 h, the *P. aeruginosa* strains began to display different growth patterns; *P. aeruginosa* strain ATCC 9027 entered the exponential phase well in advance of the other strains. *P. aeruginosa* strain RBH demonstrated the shortest exponential phase producing the lowest OD_600 nm_ (0.68 after 11.17 h incubation). The strain that generated the highest OD_600 nm_ and therefore produced the highest biomass was *P. aeruginosa* strain PAO1 (1.85 after 13.67 h incubation), followed by *P. aeruginosa* strain MRI (1.64 after 14.83 h incubation), then *P. aeruginosa* strain ATCC 9027 (1.59 after 15.83 h incubation).

The *T*_d_ (where *T*_d_ is the time required for the population to double the number of cells) and highest μ (growth rate) of each strain was calculated (Table 1). *P. aeruginosa* strain ATCC 9027 doubled in the least amount of time (*T*_d_: 2.1 h) producing the highest growth rate (μ: 0.33 h^−1^). In contrast, *P. aeruginosa* strain RBH was the slowest dividing strain in this study, producing a *T*_d_ of 3.9 h and the lowest growth rate (μ: 0.18 h^−1^).

**Table 1:** Kinetic parameters obtained from growth curves: doubling time (*T*_d_) and specific growth rate (μ) (*n* = 3).

*P. aeruginosa* strain	*T*_d_ (h)	µ (h ^−1^)
PAO1	2.9 (± 0.3)	0.24 (± 0.02)
ATCC 9027	2.1 (± 0.1)	0.33 (± 0.02)
MRI	2.7 (± 0.02)	0.26 (± 0.002)
RBH	3.9 (± 0.07)	0.18 (± 0.003)

### Antibiotic resistance profiles

**Fig. 2:**
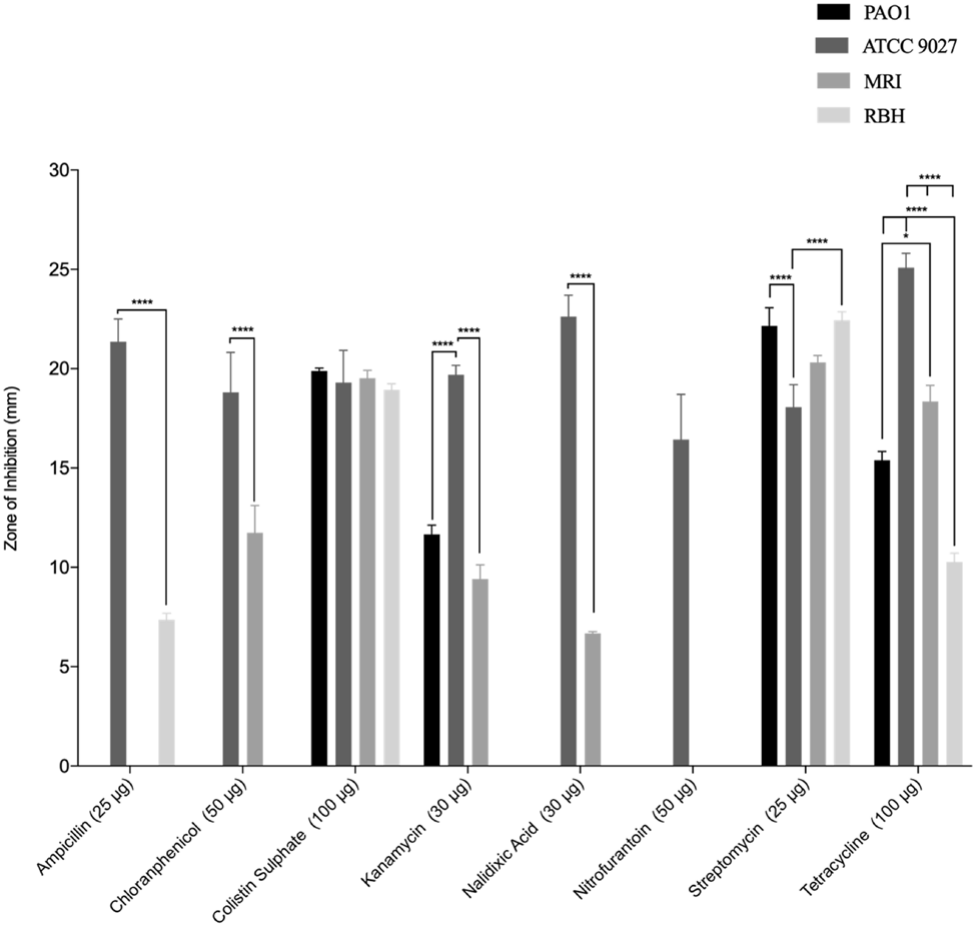
*Pseudomonas aeruginosa* antimicrobial sensitivity profiles determined by zone of inhibition measurements against a range of commonly prescribed antibiotics (*n* = 3). Asterisks denote significance, *p ≤ 0.05, **p ≤ 0.01, ***p ≤ 0.001 and ****p ≤ 0.0001

The antibiotic resistance profiles of the *P. aeruginosa* strains were determined (Fig. 2). Bacterial isolates were defined as antibiotic resistant (AMR) if they were resistant to multiple antibiotics. The least susceptible *P. aeruginosa* strains were PAO1 and RBH, both of which demonstrated resistance to four of the eight antibiotics tested. *Pseudomonas aeruginosa* strain PAO1 exhibited no sensitivity to ampicillin, chloramphenicol, nalidixic acid and nitrofurantoin. *P. aeruginosa* strain RBH showed no sensitivity to chloramphenicol, kanamycin, nitrofurantoin and nalidixic acid. *Pseudomonas aeruginosa* strains ATCC 9027 and MRI were the most susceptible, with growth inhibition observed against all of the antibiotics tested. The largest zone of inhibition observed (25.1 mm) was produced using tetracycline against *P. aeruginosa* strains ATCC 9027.

### Antimicrobial activity of ionic gold and target antibiotics

**Fig. 3:**
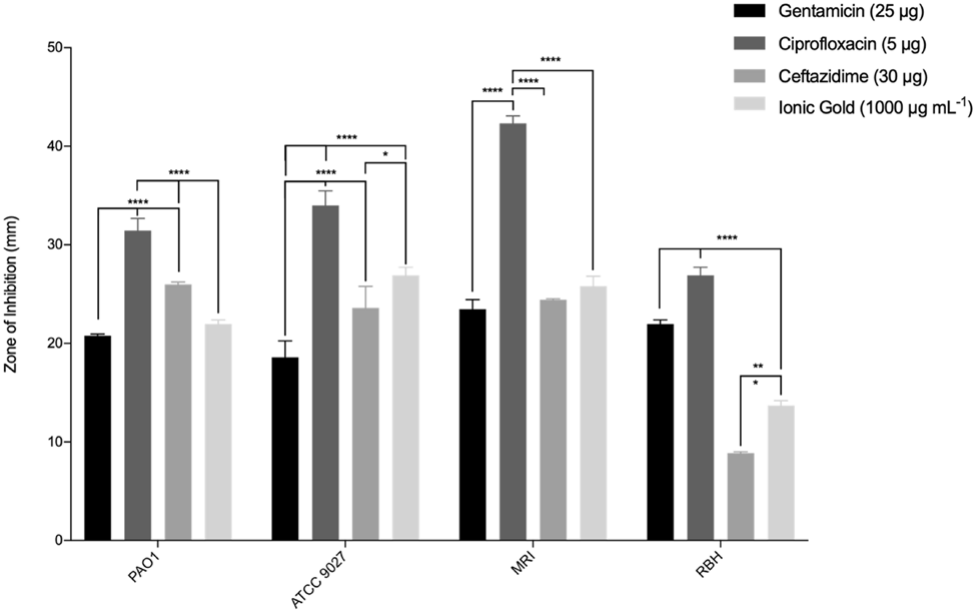
Zones of inhibition of gentamycin, ciprofloxacin, ceftazidime and ionic gold against *P. aeruginosa* strains after 18 h incubation at 37 °C (*n* = 3). Asterisks denote significance, *p ≤ 0.05, **p ≤ 0.01, ***p ≤ 0.001 and ****p ≤ 0.0001

The antimicrobial activity of the ionic gold was characterised using zone of inhibition assays and minimum inhibitory/bactericidal concentrations. The antibiotics (ciprofloxacin, ceftazidime and gentamicin) which are regularly used to treat *P. aeruginosa* infections were used as appropriate comparisons. Ionic gold demonstrated antimicrobial activity against all four *P. aeruginosa* strains (Fig. 3). Ionic gold exhibited the least antimicrobial effect against *P. aeruginosa* strain RBH, producing a ZoI of 13.7 mm and the greatest effect against *P. aeruginosa* ATCC 9027 (28.89 mm). The inhibition of the bacterial growth in the presence of ionic gold were similar between the *P. aeruginosa* strains (13.69 mm – 26.89 mm). However, although the ionic gold demonstrated antimicrobial efficacy, when compared with the antibiotics (8.88 mm – 42.33 mm), the ionic gold inhibition values were significantly lower (*p* < 0.001). Ciprofloxacin produced the largest ZoI against *P. aeruginosa* strain MRI (42.3 mm).

**Fig. 4:**
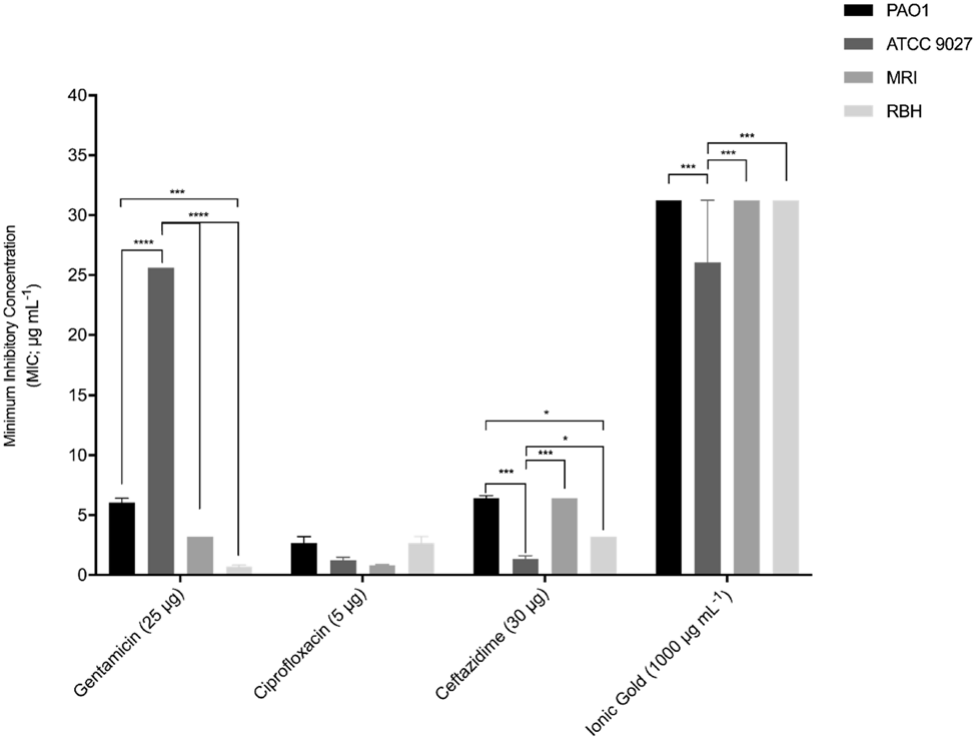
Minimal inhibitory concentrations (MIC) of gentamycin, ciprofloxacin, ceftazidime and ionic gold against *P. aeruginosa* strains after 18 h incubation (conditions: TSB, 37 °C, static) (*n* = 3). Asterisks denote significance, *p ≤ 0.05, **p ≤ 0.01, ***p ≤ 0.001 and ****p ≤ 0.0001

The MICs of ionic gold was consistent against the *P. aeruginosa* strains (26.00 µg mL^−1^ – 31.25 µg mL^−1^) (Fig. 4). The antibiotics in this study produced significantly lower MIC values than the ionic gold (0.71 μg mL^−1 ^– 25.60 μg mL^−1^) (*p* < 0.01), except for gentamycin against *P. aeruginosa* strain ATCC 9027 which exhibited a MIC of 25.60 µg mL^−1^. All three target antibiotics (gentamycin, ciprofloxacin and ceftazidime) were effective at inhibiting *P. aeruginosa* growth and ciprofloxacin was the most effective producing MIC values in the range of 0.8 µg mL^−1^ – 2.7 µg mL^−1^.

### Minimum bactericidal concentration (MBC)

**Fig. 5:**
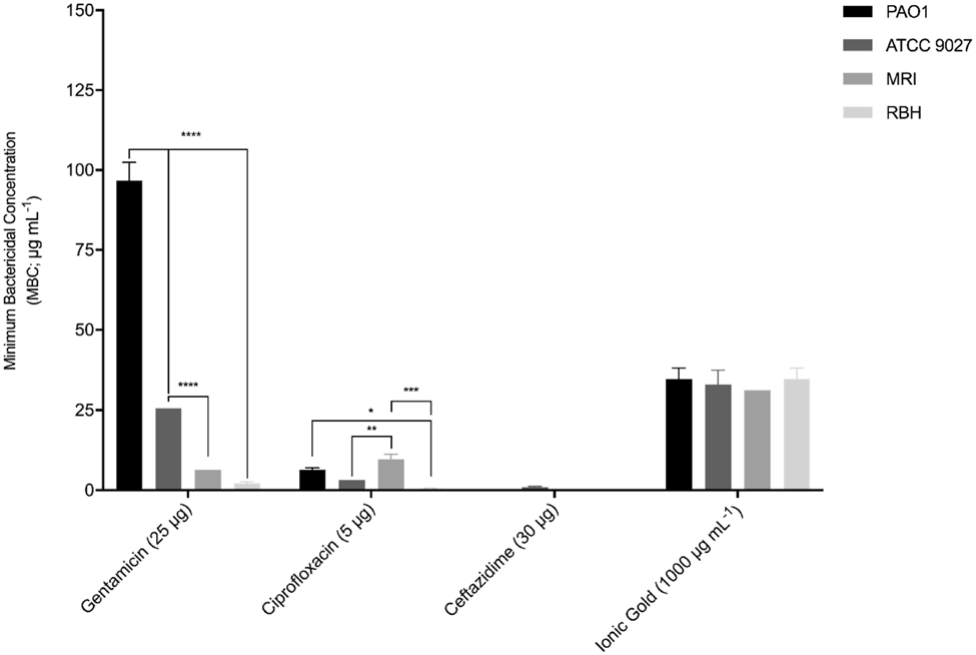
Minimal bactericidal concentrations (MBC) of gentamycin, ciprofloxacin, ceftazidime and ionic gold against *P. aeruginosa* strains after 18 h incubation (*n* = 3). Against *P. aeruginosa* strains PAO1, MRI and RBH, Ceftazidime demonstrated no bactericidal activity. Asterisks denote significance, *p ≤ 0.05, **p ≤ 0.01, ***p ≤ 0.001 and ****p ≤ 0.0001

The MBC of ionic gold and gentamycin, ciprofloxacin and ceftazidime was determined (Fig. 5). A higher concentration of ionic gold was required to eradicate the *P. aeruginosa* strains producing MBC values ranging from 31.3 µg mL^−1^ to 34.7 µg mL^−1^. The required MBC of gentamycin, ciprofloxacin and ceftazidime was lower than ionic gold except for gentamycin against *P. aeruginosa* strain PAO1 (96.7 µg mL^−1^). The most effective antimicrobial in this study was ciprofloxacin, producing the lowest MBC range, 2.7 µg mL^−1^ – 9.6 µg mL^−1^. No bactericidal activity was observed by ceftazidime against three of the four *P. aeruginosa* strains, an MBC of 0.99 µg mL^−1^ was observed against *P. aeruginosa* strain ATCC 9027, with growth observed at the maximum concentrations tested.

### Effect of ionic gold on *P. aeruginosa* cellular ultrastructure

**Fig. 6:**
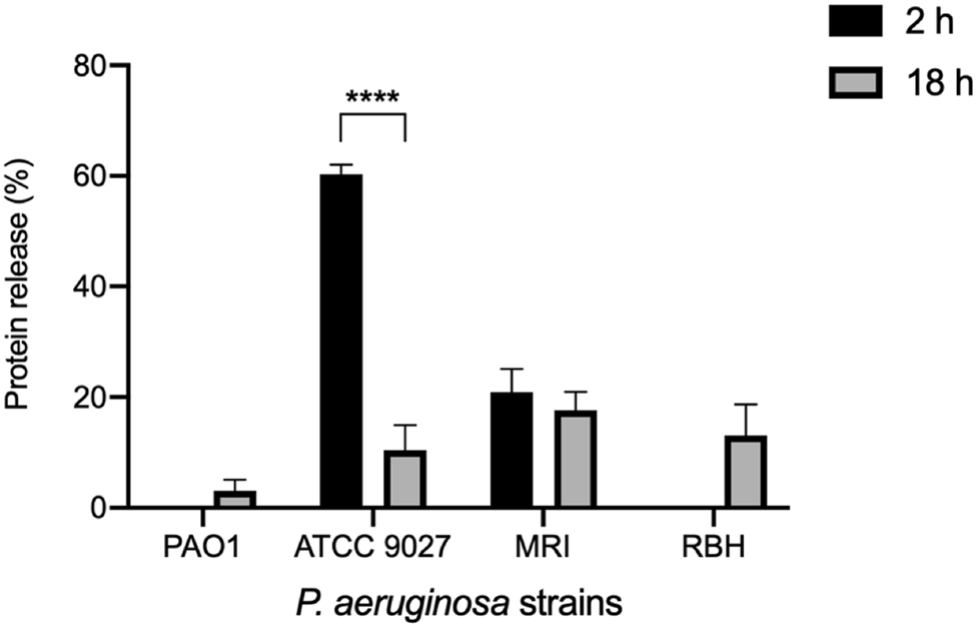
Percentage of protein released from the cytoplasm of *P. aeruginosa* due to the activity of ionic gold (*n* = 3)

Protein release into the extracellular medium was measured using the Lowry assay. Protein release was determined after two separate incubation periods, 2 h and 18 h to determine both the short- and long-term exposure effects of the ionic gold against *P. aeruginosa* (Fig. 6)*.* After short-term exposure (2 h), only two (ATCC 9027 and MRI) of the four *P. aeruginosa* strains demonstrated protein leakage from the cytoplasm into the surrounding growth media. However, after 18 h incubation with ionic gold all four *P. aeruginosa* strains exhibited protein release with *P. aeruginosa* strain MRI demonstrating the greatest protein release after 18 h (17.5%). Interestingly, the greatest protein leakage (60.3%) was determined after 2 h incubation from *P. aeruginosa* strain ATCC 9027.

### Scanning electron microscopy (SEM)

**Fig. 7:**
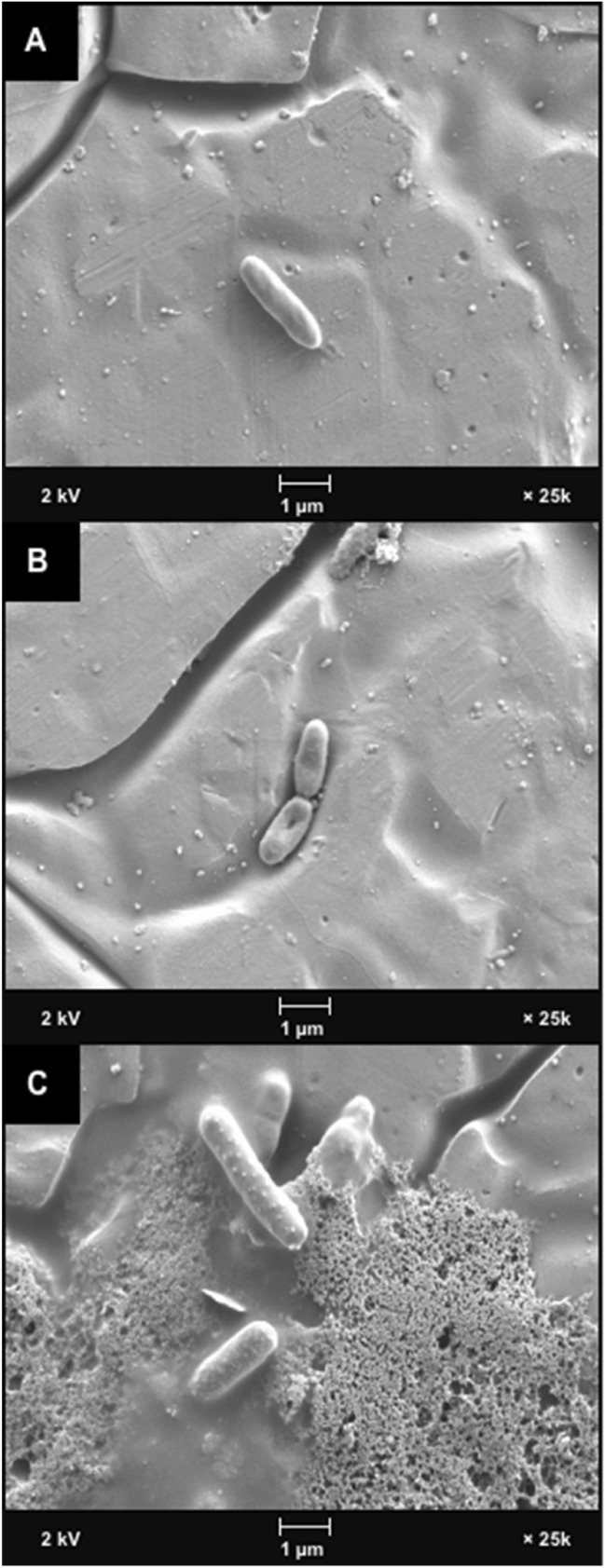
Scanning electron microscopy (SEM) of *P. aeruginosa* (**a**) PAO1 control (**b**) ionic gold treated for 2 h and (**c**) ionic gold treated for 18 h

To verify the protein leakage assays, scanning electron microscopy (SEM) was conducted, so that the antimicrobial effect of ionic gold on the *P. aeruginosa* ultrastructure could be visualised (Fig. 7). The results demonstrated that after 2 h incubation with ionic gold the cellular ultrastructure of *P. aeruginosa* strain PAO1 was compromised, this was evident by the appurtenance of defects in the cell membrane which could indicate leakage of intercellular material (Fig. 7b). After 12 h incubation, cellular debris was evident, and the *P. aeruginosa* cell morphology had altered. The intact cells demonstrated small nodules which could be observed along the surface (Fig. 7c).

## Discussion

Antimicrobial resistance (AMR) is an ever-exacerbating, global threat to human and animal health (Aslam et al. 2018). Factors such as excessive overuse of antibiotics, poor sanitation/hygiene, increased international travel and the release of non-metabolised antibiotics (or their residues) into the environment through faeces/manure, can all contribute to AMR (Ventola 2015; Aslam et al. 2018; Slate et al. 2018). Due to this, research into alternative antimicrobial therapies is of paramount importance.

The disc diffusion assays demonstrated that the ionic gold and ceftazidime exhibited similar antimicrobial effects against all the *P. aeruginosa* strains, whilst the antibiotics gentamicin and ciprofloxacin demonstrated more diverse zones of inhibition for the bacteria. Both the clinical strains demonstrated the greatest antimicrobial resistance. The difference in the growth and antibiotic resistance profiles demonstrates the need for bacterial strains from relevant environments to also be used in studies alongside type strains. The results from this study demonstrated that the clinical *P. aeruginosa* strains produced the slowest growth rate. This could be related to their antimicrobial resistance profiles, as resistance mutations are known to infer significant fitness costs (Melnyk et al. 2015).

The results for the MICs and MBCs of the ionic gold were consistent between the *P. aeruginosa* strains. The antibiotics in this study produced significantly lower MIC values than the ionic gold. However, in four cases, the MBCs of the ionic gold proved to have MBCs lower than some of the antibiotics. The antimicrobial efficacy of the ionic gold was not related to the antimicrobial resistance profiles of the bacteria, and the ionic gold demonstrated similar antimicrobial values against all the strains tested, regardless of the assay used. Thus, unlike the antibiotics, the bactericidal activity of the ionic gold was effective against all the *P. aeruginosa* strains regardless of their antibiotic resistance profile. Although ionic gold demonstrated lower antimicrobial activity than commonly used antibiotics against *P. aeruginosa*, the multiple antimicrobial mechanisms which occur simultaneously, may result in less resistance generation (Lansdown 2002; Gold et al. 2018). This suggests that when antibiotic use is not viable due to resistance concerns, the use of an alternative antimicrobials such as ionic gold may be one alternative approach.

Gold is a rare, inert and non-essential heavy metal to bacteria (Srivastava et al. 2013; Wyatt et al. 2014). Ionic gold in the form of Au^+^ and Au^3+^ has known antimicrobial activity (Zhang et al. 2015). Few studies have demonstrated the antimicrobial efficacy of ionic gold; however, a plethora of studies have demonstrated antimicrobial activity of gold nanoparticles and gold containing compounds (such as auranofin) (Li et al. 2014; Thangamani et al. 2016; Vaidya et al. 2017). In 2015, Dasari et al., demonstrated the antibacterial activity of ionic gold against four different bacteria, including one non-pathogenic bacterial species, *Escherichia coli* and three MDR bacterial species’: *E. coli*, *Salmonella typhiurium* and *Staphylococcus aureus* (Dasari et al. 2015). Both Au^+^ and Au^3+^ were highly toxic to all four bacteria, with Au^+^ producing an IC_50_ (half maximal inhibitory concentration) range of 0.35 µM – 0.49 µM, whilst Au^3+^ produced an IC_50_ range of 0.27 µM – 0.52 µM (Dasari et al. 2015). In a recent study by Karaky et al (2020), ionic gold produced a MIC of 26 µg mL^−1^ and a MBC of 41.7 µg mL^−1^ against two clinical isolates of *P. aeruginosa* (Karaky et al. 2020).

Despite the known antimicrobial activity of ionic gold, the mechanism of action is yet to be fully elucidated. Like other metal ions, the antimicrobial activity is hypothesised to involve multiple mechanisms (Lemire et al. 2013; Wang et al. 2016). Such antimicrobial mechanisms are thought to include the generation of reactive oxygen species (ROS) and the depletion of antioxidants. In addition, metal ions can bind to the thiol groups of proteins and enzymes leading to dysfunction (Feng et al. 2000; Lemire et al. 2013; Stiefel et al. 2015). These mechanisms can damage cellular membranes, disrupt electron transport and interfere with nutrient acquisition, ultimately resulting in cell lysis and death (Sondi and Salopek-Sondi 2004; Hobman and Crossman 2015). Furthermore, metal ions can also intercalate with the phosphorous elements of bacterial DNA, resulting in an impaired ability to replicate (Kim et al. 2007). Although resistance to metal ions has been observed previously (Lemire et al. 2013), it is less likely to be effective at completely inhibiting the antimicrobial activity when compared with traditional antibiotics, due to the multiple mechanisms of cellular disruption involved, simultaneously. Throughout this study, *P. aeruginosa* protein leakage into the extracellular media due to the presence of ionic gold was demonstrated using a protein quantification assay (Lowry assay) and SEM visualisation, and it was evident that all four *P. aeruginosa* strains demonstrated cellular leakage and damage. This suggests that damage to the cellular ultrastructure was in part responsible for the antimicrobial activity of ionic gold.

For ionic gold to be applied as a topical agent or as part of a wound dressing its cytotoxicity towards relevant mammalian cells must be considered. The cytotoxicity of gold ions and gold nanoparticles is poorly understood with studies suggesting controversial results throughout the literature (Alkilany and Murphy 2010; Barbasz and Oćwieja 2016; Falagan-Lotsch et al. 2016; Gunduz et al. 2017). Ionic gold is a powerful inhibitor of macrophages and polymorphonuclear leucocytes. However, most reports focus on the release of ionic gold from prototype implants and cellular accumulation (Hostynek 1997; Danscher 2002). However, Barbasz et al*.,* (2016) determined that gold ions had no significant antimicrobial activity on either a human monocytic (U-937) or a human promyelocytic leukaemia cell line, demonstrating a 9% and 12% reduction in cell survival, respectively, when compared with the control (Barbasz and Oćwieja 2016). The presence of gold ions although deemed non-toxic, has been shown to enhance nitric oxide concentrations fivefold, indicating an inflammatory response (Barbasz and Oćwieja 2016). To progress ionic gold as a topical ointment, wound dressing or implant coating further research is required in determining both the mode of antimicrobial action of ionic gold and its effect on mammalian cytotoxicity.

## Conclusions

Throughout this study, the antimicrobial activity of ionic gold was evaluated against four *P. aeruginosa* strains and were compared against commonly prescribed antibiotics. Both the clinical strains demonstrated the greatest antimicrobial resistance profiles. The difference in the growth and antibiotic resistance profiles demonstrates the need for bacterial strains from relevant environments to also be used in studies alongside type strains. The clinical bacterial strains of *P. aeruginosa* demonstrated they were the slowest at dividing and this could be related to their antimicrobial resistance profiles. Throughout the MBC assays, ionic gold demonstrated good bactericidal activity against the *P. aeruginosa* strains. The antimicrobial efficacy of the ionic gold was not related to the antimicrobial resistance profiles of the bacteria, and the ionic gold demonstrated similar antimicrobial values for all the strains tested, regardless of the assay used. Although ionic gold demonstrated lower antimicrobial activity against *P. aeruginosa*, when compared with the antibiotics which are commonly utilised, the multiple antimicrobial mechanisms which occur simultaneously, may result in less resistance generation. The results from this study indicated that protein leakage was apparent, upon incubation with ionic gold, demonstrating damage to the *P. aeruginosa* cell ultrastructure. Therefore, the utilisation of ionic gold as an antimicrobial topical agent, or as part of a wound dressing or implant coating may be potential avenues to be explored to reduce excessive antibiotic use.

## Data Availability

The datasets used and/or analysed during the current study are available from the corresponding author on reasonable request.
